# Key role of reactive oxygen species-scavenging system in nitric oxide and hydrogen sulfide crosstalk-evoked thermotolerance in maize seedlings

**DOI:** 10.3389/fpls.2022.967968

**Published:** 2022-11-07

**Authors:** Yu-Ying Sun, Jia-Qi Wang, Ru-Hua Xiang, Zhong-Guang Li

**Affiliations:** ^1^ School of Life Sciences, Yunnan Normal University, Kunming, China; ^2^ Engineering Research Center of Sustainable Development and Utilization of Biomass Energy, Ministry of Education, Kunming, China; ^3^ Key Laboratory of Biomass Energy and Environmental Biotechnology, Yunnan Province, Yunnan Normal University, Kunming, China

**Keywords:** nitric oxide, hydrogen sulfide, reactive oxygen species, reactive oxygen species-scavenging system, maize seedlings

## Abstract

Nitric oxide (NO) and hydrogen sulfide (H_2_S) are novel signaling molecules, which participate in plant growth, development, and response to stress. In this study root-irrigation with 0.15 mM sodium nitroprusside (SNP, NO donor) up-regulated gene expression of *L-CYSTEINE DESULFHYDRASE1* (*LCD1*), activities of L-cysteine desulfhydrase (LCD) and D-cysteine desulfhydrase (DCD), as well as an endogenous H_2_S level, compared to control seedlings. The SNP-up-regulated effects were enhanced by 0.5 mM sodium hydrosulfide (NaHS, H_2_S donor), but weakened by NO scavenger 2-(4-carboxyphenyl)-4,4,5,5-tetramethylimidazoline-1-oxyl-3-oxide (cPTIO) and H_2_S scavenger hypotaurine (HT) alone. NaHS had no significant effect on gene expression and activity of nitrate reductase (NR, a NO candidate producing enzyme). These data indicate that NO could trigger the LCD/H_2_S signaling pathway in maize seedlings. To further investigate the effect of NO and H_2_S crosstalk on thermotolerance in maize seedlings, thermotolerance parameters and reactive oxygen species (ROS)-scavenging system were estimated. The results show that SNP increased survival rate and tissue viability, decreased malondialdehyde (MDA) accumulation, and electrolyte leakage in maize seedlings under heat stress (HS), implying NO could improve thermotolerance in maize seedlings. The NO-improved thermotolerance was impaired by H_2_S inhibitor DL-propargylglycine (PAG) and scavenger HT alone. Similarly, SNP up-regulated the gene expression of *DEHYDROASCORBATE REDUCTASE* (*DHAR)* and *GLUTATHIONE REDUCTASE1* (*GR1*); activities of ascorbate peroxidase, glutathione reductase, and catalase; as well as levels of ascorbic acid, glutathione, flavonoids, carotenoids, and total phenols. SNP also reduced hydrogen peroxide and superoxide radical accumulation in maize seedlings under HS compared to the control. The effects of SNP on ROS and their scavenger system were weakened by PAG and HT alone. These data hint that NO could evoke thermotolerance in maize seedlings by triggering the LCD/H_2_S signaling pathway, and the ROS-scavenging system played a key role in the NO and H_2_S crosstalk-evoked thermotolerance.

## Introduction

Nitric oxide (NO) is an important signaling molecule in plants, it can be produced by nitrate reductase (NR) using nitrite as a substrate (Bruand and Meilhoc, 2019; Kolbert et al., 2019; Gupta et al., 2022; Singh et al., 2022). Mounting evidence shows that NO plays a key role in seed germination, seedling emergence, plant blooming, fruit fresh-keeping, and plant response to abiotic stresses (such as heat, salt, drought, heavy metal, and flooding stresses) by interacting with signaling molecule hydrogen sulfide (H_2_S) (Liu et al., 2012; Wang et al., 2012; Shi et al., 2014; Peng et al., 2016; Muñoz-Vargas et al., 2018; Sun et al., 2018; Xie et al., 2018; Mukherjee, 2019). In rice seedlings, NO application could enhance thermotolerance by modulating antioxidant defense, carbohydrate metabolism, and photosynthesis *via* the interplay with ethylene, H_2_S, and sulfur (Gautam et al., 2022a; Gautam et al., 2022b; Sehar et al., 2022). Similarly, in wheat seedlings, foliar application of NO alone or in combination was able to improve photosynthesis under heat stress by reducing H_2_O_2_-induced oxidative stress and excess glucose-mediated photosynthetic suppression (Iqbal et al., 2021). Our previous study also found that NO-H_2_S interaction triggered thermotolerance in maize seedlings (Li et al., 2013), but the underlying mechanisms remain unclear.

H_2_S is one of the gaseous signaling molecules (such as NO, carbon monoxide, ethylene, ammonia, and hydrogen), and plays a critical role in many physiological processes in plants under optimal and stress conditions (Li et al., 2016; Ahmed et al., 2021). H_2_S homeostasis in plant cells can be modulated by LCD/DCD, O-acetylserine (thio) lyase (OAS-TL), and sulfite reductase (SiR) (Liu et al., 2021; Mishra et al., 2021; Singh et al., 2021). These enzymes and H_2_S have a leading role in numerous physiological processes from seed germination to plant senescence. As mentioned above, H_2_S can not only exert its physiological functions alone (Li et al., 2016; Liu et al., 2021) but also regulates the acquirement of stress tolerance including thermotolerance in plants by interacting with NO (Li et al., 2013; Xie et al., 2018; Huang et al., 2021; Li et al., 2021; Gautam et al., 2022a; Mishra et al., 2022; Sehar et al., 2022). However, the exact interaction mechanism between H_2_S and NO in plants is not completely clear.

In general, plant cells inevitably produce ROS (mainly superoxide radical, O_2_^.-^; hydrogen peroxide, H_2_O_2_) by enzymatic and non-enzymatic pathways. This production is enhanced under stress conditions including heat stress (HS) (Yadav et al., 2018; Wani and Kumar, 2020). With the development of global warming, HS has become a principal abiotic stress factor determining plant productivity, accompanying the whole life cycle of plants (Saidi et al., 2011; Hasanuzzaman et al., 2013). HS commonly leads to oxidative damage due to the over-accumulation of ROS. To maintain ROS homeostasis in plant cells, the level of ROS is strictly controlled by the ROS-scavenging system, which is composed of enzymatic and non-enzymatic antioxidants (Szymanska et al., 2017). The enzymatic antioxidants mainly incorporate ascorbate peroxidase (APX), dehydroascorbate reductase (DHAR), monodehydroascorbate reductase (MDHAR), glutathione reductase (GR), superoxide dismutase (SOD), catalase (CAT), and peroxidase (POD). The non-enzymatic antioxidants chiefly refer to ascorbic acid (AsA), glutathione (GSH), flavonoids (FLA), carotenoids (CAR), and polyphenols (Ashraf, 2021; Haider et al., 2021). Under both physiological and stress conditions, the ROS-scavenging system has a leading role in many physiological processes in plants to maintain ROS homeostasis or trigger ROS signaling (Wahid et al., 2007; Zhao et al., 2020). Therefore, the enhancement of the ROS-scavenging system is a positive correlation with plant stress tolerance including thermotolerance.

Maize is the third cereal crop and new model plant, the seedling stage is the critical period to determine yield and field harvest due to its sensitivity to HS (Strable and Scanlon, 2009; Tiwari and Yadav, 2019). Therefore, how to improve the thermotolerance of maize seedlings and resolve its underlying mechanisms is an urgent issue. In this paper, the NO and H_2_S crosstalk-evoked thermotolerance and the underlying mechanisms in maize seedlings were dissected. It was designed to illustrate the key role of the ROS-scavenging system in the NO and H_2_S crosstalk-evoked thermotolerance.

## Materials and methods

### Seed germination and seedling culture

Maize (*Zea mays* L., cv. Diwo No. 2) seeds were purchased from Shiling Seed Company, China. The healthy and uniform size seeds were sterilized in 0.1% HgCl_2_ solution for 10 min and then rinsed seven times with distilled water to wash off the residual HgCl_2_. The rinsed seeds were immersed in distilled water for 12h to imbibe. The watered seeds were sown on the wetted filter papers in plates with covers (about 200 seeds per plate) and germinated in the dark for 60 h in a climate chamber with 26°C and relative humidity (RH) of 65% ± 5%. The 2-cm high seedlings (i.e. 60-h-old seedlings) were irrigated with the following chemicals and HS.

### Chemical root-irrigation and HS treatments

The 2-cm high seedlings were divided into 14 groups and irrigated with different chemicals for 4 h (appropriate time was derived from preliminary experiments, the equivalent effect could be gained from 4 to 8 h of irrigation). (1) Distilled water (CK), (2) 0.5 mM NaHS, (3) 0.15 mM SNP, (4) 0.3 mM HT, (5) 0.15 mM cPTIO, (6) 0.5 mM NaHS + 0.15 mM SNP, (7) 0.15 mM cPTIO + 0.5 mM NaHS, (8) 0.15 mM methylene blue (MB) + 0.5 mM NaHS, (9) 0.15 mM N-ω-nitro-L-arginine (NNA) + 0.5 mM NaHS, (10) 0.15 mM sodium tungstate (ST) + 0.5 mM NaHS, (11) 0.3 mM PAG + 0.15 mM SNP, (12) 0.3 mM hydroxylamine (HA) + 0.15 mM SNP, (13) 0.3 mM sodium pyruvate (SP) + 0.15 mM SNP, (14) 0.3 mM HT + 0.15 mM SNP. Among these chemicals, NaHS and HT are H_2_S donors and scavengers, respectively, while PAG, HA, and SP are H_2_S inhibitors (Li et al., 2013; Singh et al., 2021). Similarly, SNP, cPTIO, and MB are NO donors and scavengers, respectively, while NNA and ST are NO inhibitors (Singh et al., 2022). The suitable concentrations of these chemicals were rooted in preliminary experiments and our precious studies (Li et al., 2013; Zhou et al., 2018; Ye et al., 2020). To acquire a better effect, in the scavenger and inhibitor experiments, the 58-h-old seedlings were pre-irrigated with scavengers or inhibitors for 2 h and then irrigated with the second chemical for 4 h (e.g. 0.15 mM cPTIO + 0.5 mM NaHS). In the other combined experiments, to avoid chemical reaction *in vitro*, the 56-h-old seedlings were pre-irrigated with the first chemical for 4 h and then irrigated with the second chemical for 4 (e.g. 0.5 mM NaHS + 0.15 mM SNP). The non-irrigated (CK) and chemical-irrigated seedlings were subjected to HS at 46°C for 16 h in the dark in a climate chamber (this temperature was a half-lethal strength, and the survival rate of the control seedlings irrigated with distilled water was approximately 50%) with an RH of 65% ± 5%. After chemical and HS irrigations, the buds of maize seedlings were sampled to estimate the following physiological and molecular parameters due to their sensitivity to HS among seedling organs (Li et al., 2013; Wang et al., 2019).

### Quantification of NO, H_2_S, and their metabolic enzyme activity and gene expression

After chemical and HS irrigations, the contents of H_2_S and NO in the buds (aboveground parts) of maize seedlings were determined using the methods reported (Li et al., 2015; Shahi and Srivastava, 2018; Ye et al., 2020). Correspondingly, the contents of soluble proteins in buds of maize seedlings were measured using the Bradford method (Bradford, 1976) using bovine serum albumin as a standard sample. Their activities were expressed in nmol min^-1^ mg^-1^ protein, while H_2_S and NO contents were expressed as a nmol g^-1^ fresh weight (FW). In addition, the expression of *LCD1*, *OAS-TL*, and *NR* was detected by qRT-PCR (using *Zea mays* beta-5 tubulin (*ZmTUB*) as a reference gene) (Qiu et al., 2022), and the primer information of these genes is listed in **Table 1**.

**Table 1:** Genes and primer information was used in this study.

Gene	Accession number	Primer Sequence (5´→3´)
*ZmTUB*	NM_001111988	F:AGAACTGCGACTGCCTCCAAAGG R:AGATGAGCAGGGTGCCCATTC
*ZmLCD1*	NM_001138259	F:AAGTGTTGAGGAAGGACAAGAG R:GGCATCTCTCAAGACCTCATAC
*ZmOAS-TL*	NM_001366967	F:GGCAAGTACCTCAAGGAGAAA R:CTACTCCGTTTCCAGTGATGAG
*ZmNR*	NM_001305856	F:CCAGCGTAAATTTCGTGAGATG
		R: TGCTGCTCTAGTCTGGTAATTC
*ZmCAT1*	NM_001254879.2	F:GGGTCCAGACACCTGTTATTG
		R:AGTTACCCTCTCTGGTGTAGAA
*ZmSOD4*	NM_001112234.2	F:CGTCACCAGCAGGCTAGAAT
		R:AGCCAACAGTCCAACACAGT
*ZmGR1*	NM_001305818.1	F:CTCTCACGAGTTTGAAGAGTCTCGTGG
		R:CCAGCGCAGCATCCGAATCTATAA
*ZmAPX1*	NM_001370758.1	F:GATCTTGTGGCTGCAGCATG
		R:GGTGGACTCGAATTGCAGGA
*ZmMDHAR*	NM_001196274.1	F:AAGTGGTGGAGAGAAGCTATTG
		R:CTAGTCAGAGTCTTGGTGGAAAG
*ZmDHAR1*	NM_001147572.1	F:ATCTCTGGTCACTCCTGTAGAA
		R:CTCGGAACCATCACTAGCATC

### HS and estimation of thermotolerance parameters

After HS, the heated seedlings were cultured at 26°C in a climate chamber with 200 µmol·m^-2^·s^-1^, 14 h/10 h (day/night) photoperiod, and RH of 65% ± 5% for seven days and applied fertilizer with 1/2 Hoagland solution to recover growth. After recovery, the survival rate (SR) was estimated as the formula: SR (%) = the number of the survived seedlings/number of the total seedlings × 100%. Meanwhile, after HS, tissue viability (A_485_, i.e. triphenyl tetrazolium chloride reduction), MDA content, and electrolyte leakage (EL) were estimated as per the methods described by Wang et al. (2019). The tissue viability, MDA content, and EL were expressed in A_485_, μmol·g^-1^ FW, and %.

### Enzymatic antioxidant activity and gene expression assay

After chemical and HS irrigations, the enzymatic antioxidants (i.e. APX, DHAR, MDHAR, GR, CAT, and SOD) in buds of maize seedlings were extracted and estimated in the light of the previous procedures (Li, 2019; Wang et al., 2019). The soluble protein contents were assayed as per the abovementioned method (Bradford, 1976). Their activities were calculated using the extinction coefficients of 2.8 (for AsA to calculate APX), 14.0 (for AsA to calculate DHAR and MDHAR), 6.2 (for NADPH to calculate GR), and 40 (for H_2_O_2_ to calculate CAT) mM^-1^ cm^-1^ except SOD using activity unit (i.e. a unit activity refers to the amount of enzyme which inhibits 50% photochemical reduction of nitroblue tetrazolium) and expressed in nmol min^-1^ mg^-1^ protein or U mg^-1^ protein for SOD. The expression of *APX1*, *DHAR*, *MDHAR*, *GR1*, *CAT1*, and *SOD4* was detected by qRT-PCR (using *Zea mays* beta-5 tubulin (*ZmTUB*) as reference gene) (Qiu et al., 2022), the primer information of these genes was listed in **Table 1**.

### Non-enzymatic antioxidant evaluation

After chemical and HS irrigations, the contents of GSH, oxidized GSH (GSSG), AsA, oxidized AsA (DHA), FLA, CAR, and total phenols (TP) in buds of maize seedlings were extracted and evaluated as per the procedure reported by Wang et al. (2019). The contents of AsA, DHA, GSH, GSSG, and FLA were expressed in μmol g^-1^ FW, while CAR and TP were expressed as μg g^-1^ FW and nmol g^-1^ FW, respectively.

### O_2_^.-^ and H_2_O_2_ measurement

After chemical and HS irrigations, the O_2_^.-^ production rate and H_2_O_2_ content were measured using Na,3′-[1-[(phenylamino)-carbonyl]-3,4-tetrazolium] (4-metho- xy-6-nitro) benzene sulfonic acid hydrate (XTT) method and titanous sulfate method, respectively (Li, 2019). The O_2_.production rate and H_2_O_2_ content were counted as the extinction coefficient of 21.6 and 0.28 μM^-1^ cm^-1^ and expressed as nmol min^-1^ g^-1^ FW and μmol g^-1^ FW, respectively.

### Statistical analysis

The experiments involved a completely random design and the data had at least three biological replicates using Duncan's multiple-range test at a 0.05 significant level. In the figures, the data denote means ± standard error (SE), the bars with the different letters represent significant differences, while the same letters represent no significant difference.

## Results

### NO triggers H_2_S signaling in maize seedlings

**Figure 1:**
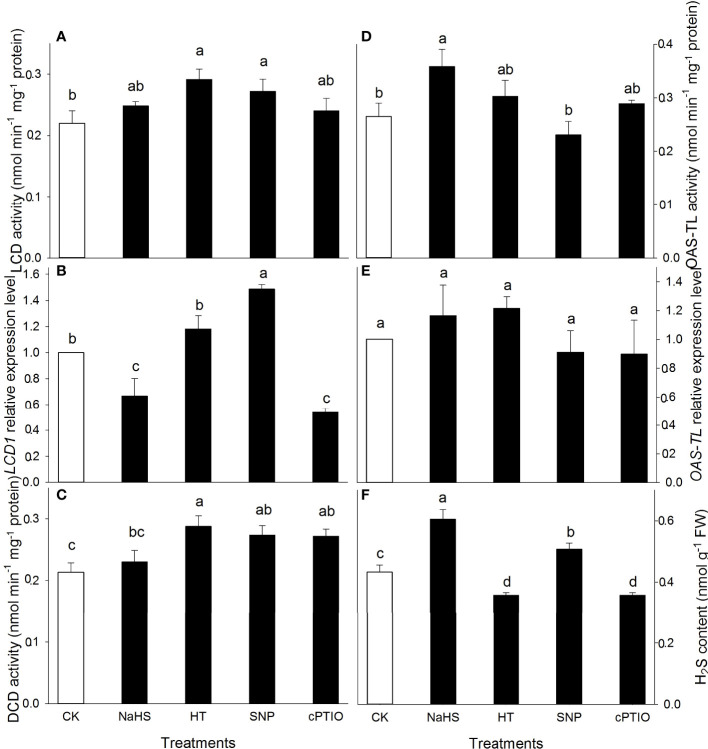
Effect of sodium hydrosulfide (NaHS), hypotaurine (HT), sodium nitroprusside (SNP), and 2-(4-carboxyphenyl)-4,4,5,5-tetramethylimidazoline-1- oxyl-3-oxide (cPTIO) on L-cysteine desulfhydrase (LCD) **(A)**, D-cysteine desulfhydrase (DCD) **(C)**, O-acetylserine (thio) lyase (OAS-TL) **(D)** activities and corresponding gene expression **(B, E)** as well as hydrogen sulfide (H_2_S) content **(F)** in maize seedlings under non-HS conditions. The data had at least three biological replicates and were tested using Duncan's multiple-range test at 0.05 significant level, which denote means ± standard error (SE). The bars with the different letters represent significant differences, while the same letters represent no significant difference.

To explore NO and H_2_S crosstalk, the activities of enzymes (LCD, DCD, and OAS-TL), expression of genes (*LCD1* and *OAS-TL*), and contents of NO and H_2_S in maize seedlings irrigated with distilled water (CK), NaHS, HT, SNP, and cPTIO were estimated. The results show that HT, SNP, and cPTIO alone significantly increased LCD (except for cPTIO) and DCD activities in maize seedlings, while OAS-TL merely increased by NaHS, compared to the CK (**Figures 1A, C, D**). Correspondingly, the gene expression of *LCD1* was remarkably up-regulated by SNP and down-regulated by NaHS and cPTIO alone. HT had no significant effect on *LCD1* expression in maize seedlings (**Figure 1B**). WE did not observe that NaHS, HT, SNP, and cPTIO alone significantly affected *OAS-TL* expression compared with the CK (**Figure 1E**). As expected, the H_2_S content in maize seedlings was markedly elevated by NaHS and SNP alone, but it was observably weakened by HT and cPTIO (**Figure 1F**). These data imply that exogenous NO could increase endogenous H_2_S content in maize seedlings.

**Figure 2:**
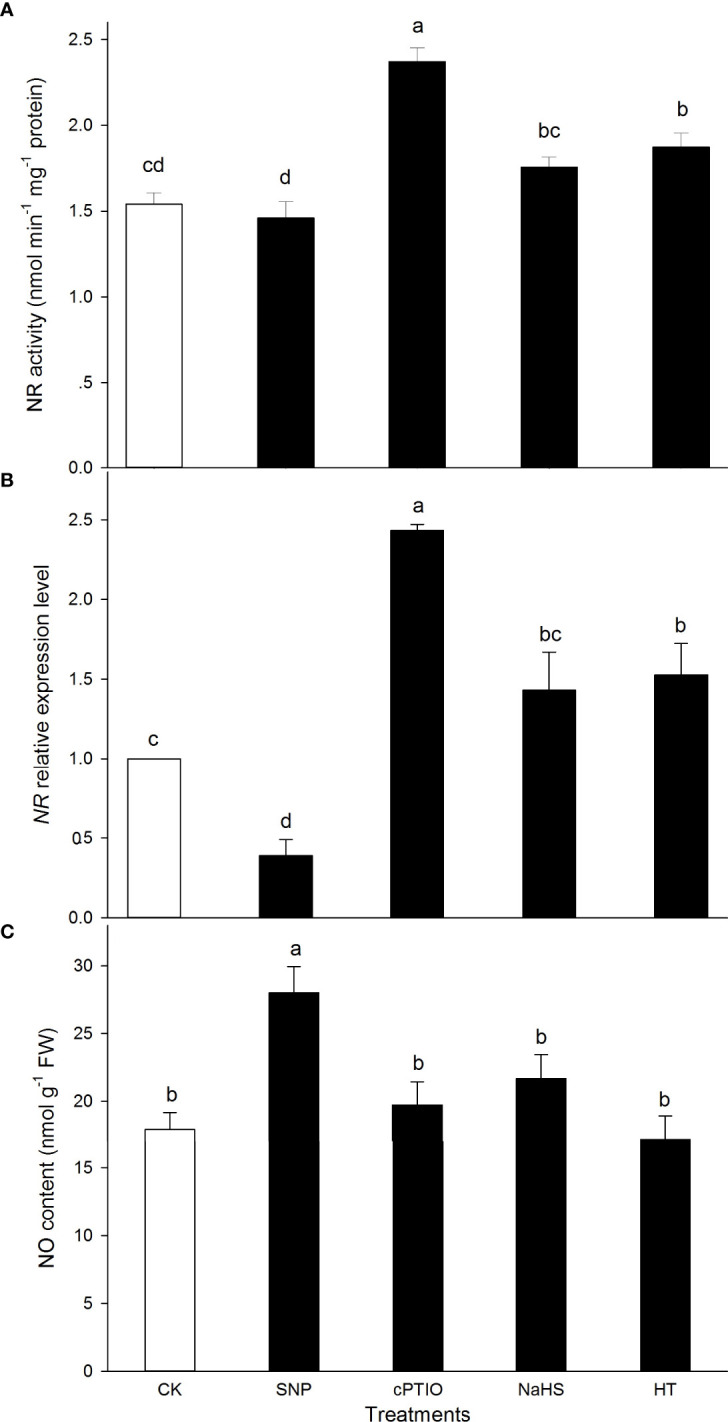
Effect of sodium nitroprusside (SNP), 2-(4-carboxyphenyl)-4,4,5,5- tetrameth- ylimidazoline-1-oxyl-3-oxide (cPTIO), sodium hydrosulfide (NaHS), and hypotaurine (HT) on nitrate reductase (NR) **(A)** activity, corresponding gene expression **(B)**, and nitric oxide (NO) content **(C)** in maize seedlings under non-HS conditions. The data had at least three biological replicates and were tested using Duncan,s multiple-range test at 0.05 significant level, which denote means ± standard error (SE). The bars with the different letters represent significant differences, while the same letters represent no significant difference.

Similarly, in comparison to the CK, the activity of NR was significantly increased by HT and cPTIO alone, while NaHS and SNP alone had no significant effect on NR (**Figure 2A**). Correspondingly, *NR* expression was observably up-regulated by HT and cPTIO alone, and down-regulated by SNP. NaHS had no significant effect on *NR* expression in maize seedlings compared with the CK (**Figure 2B**). As might be expected, the endogenous NO content in maize seedlings was significantly increased by SNP. NaHS, HT, and cPTIO on NO did not significantly affect maize seedlings (**Figure 2C**). These data indicate that exogenous H_2_S had no significant effect on endogenous NO content in maize seedlings.

### NO and H_2_S crosstalk evokes thermotolerance in maize seedlings

**Figure 3:**
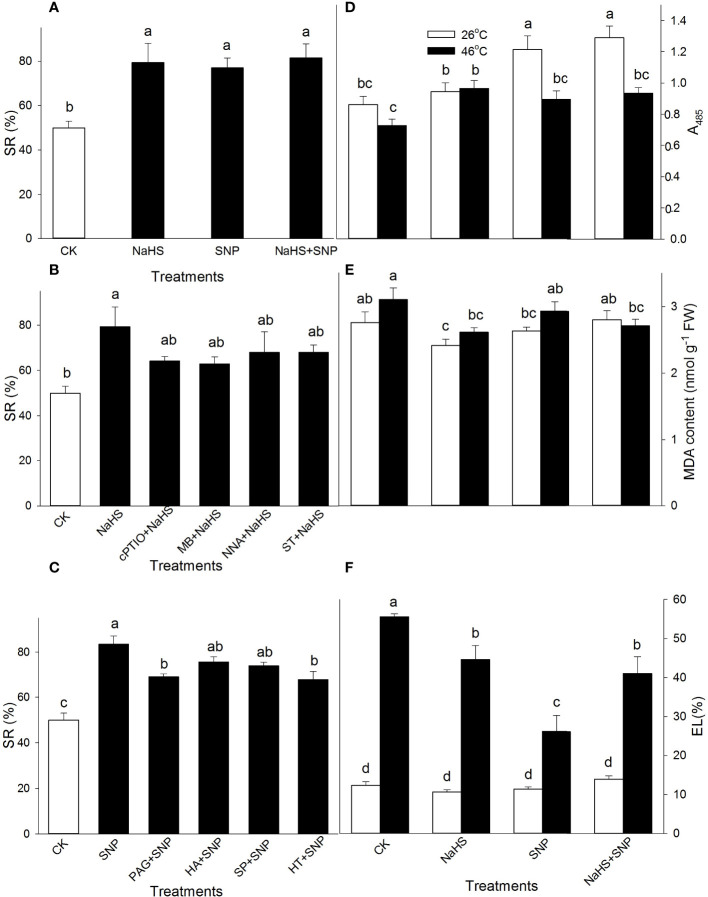
Effect of sodium hydrosulfide (NaHS) alone, or in combination with sodium nitroprusside (SNP), nitric oxide (NO) scavengers and inhibitors as well as SNP alone or in combination with hydrogen sulfide (H_2_S) inhibitors and scavengers on survival rate (SR) **(A–C**), tissue viability **(D)**, malondiadehyde (MDA) **(E)**, and electrolyte leakage (EL) **(F)** in maize seedlings under non-HS and HS conditions. The data had at least three biological replicates and were tested using Duncan's multiple-range test at 0.05 significant level, which denote means ± standard error (SE). The bars with the different letters represent significant differences, while the same letters represent no significant difference.

To further investigate the effect of NO and H_2_S crosstalk on thermotolerance in maize seedlings, the seedlings were irrigated with NaHS and SNP alone or in combination; NaHS alone or combined with cPTIO, MB, NNA, or ST; as well as SNP alone or combined with PAG, HA, SP, or HT prior to HS. As shown in **Figure 3**, compared with the CK, the SR of maize seedlings was improved by NaHS and SNP alone or in combination after HS. The significant effect of cPTIO, MB, NNA, and ST on the NaHS-improved SR was not observed (**Figure 3B**). PAG and HT significantly impaired the SNP-improved SR, but HA and SP had no significant effect on this improvement (**Figure 3C**).

Interestingly, in comparison with the CK under non-HS conditions, NaHS and SNP alone or in combination improved the tissue viability except for in the case of NaHS alone (A_485_, **Figure 3D**) and had no significant effect on MDA accumulation (except for NaHS alone) (**Figure 3E**) and EL (**Figure 3F**). Under HS conditions, in comparison to the control, NaHS alone significantly increased A_485_, while SNP alone or in combination with NaHS was no significant effect (**Figure 3D**). Similarly, NaHS alone or in combination with SNP obviously reduced MDA accumulation, but SNP had no significance (**Figure 3E**). The HS-induced increase in EL was observably alleviated by NaHS and SNP alone or in combination compared with the CK (**Figure 3F**). These data show that H_2_S acted as signaling role in the downstream of NO in the development of the plant thermotolerance.

### NO and H_2_S crosstalk up-regulates the activity and gene expression of enzymatic antioxidants

**Figure 4:**
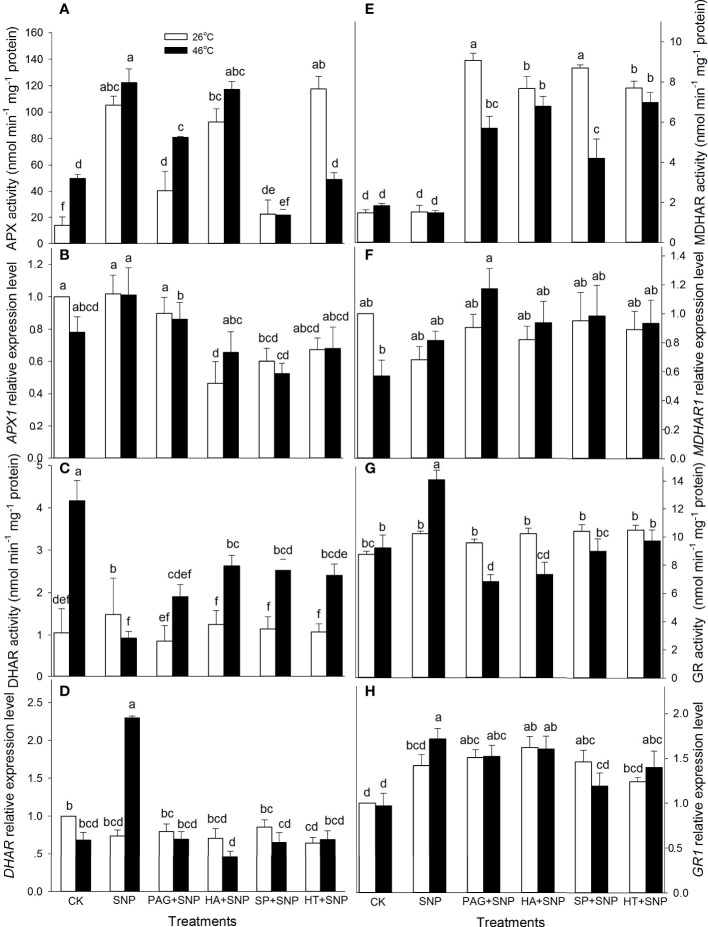
Effect of SNP alone or in combination with hydrogen sulfide (H_2_S) inhibitors and scavengers on ascorbate peroxidase (APX) **(A)**, dehydroascorbate reductase (DHAR) **(C)**, monodehydroascorbate reductase (MDHAR) **(E)**, and glutathione reductase (GR) **(G)** activities and corresponding gene expression **(B, D, F, H)** in maize seedlings under non-HS and HS conditions. The data had at least three biological replicates and were tested using Duncan's multiple-range test at 0.05 significant level, which denote means ± standard error (SE). The bars with the different letters represent significant differences, while the same letters represent no significant difference.

**Figure 5:**
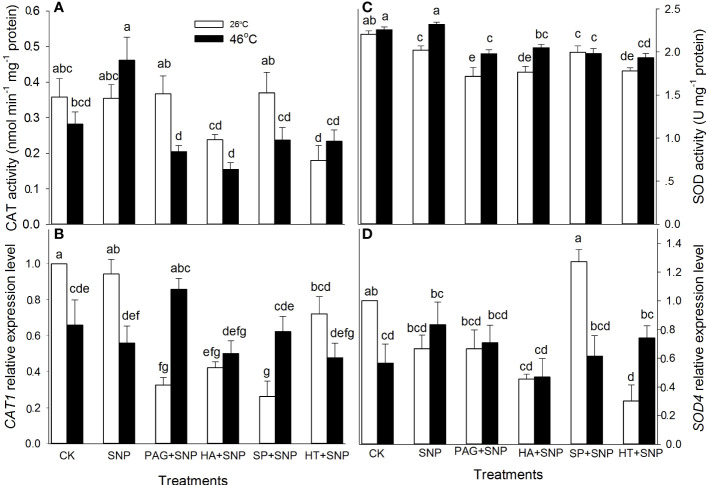
Effect of SNP alone or in combination with hydrogen sulfide (H_2_S) inhibitors and scavengers on catalase (CAT) **(A)** and superoxide dismutase (SOD) **(C)** activities and corresponding gene expression **(B, D)** in maize seedlings under non-HS and HS conditions. The data had at least three biological replicates and were tested using Duncan's multiple-range test at 0.05 significant level, which denote means ± standard error (SE). The bars with the different letters represent significant differences, while the same letters represent no significant difference.

As mentioned above, NO and H_2_S crosstalk could evoke thermotolerance in maize seedlings (**Figure 3**), to further illustrate the role of enzymatic antioxidants in the formation of thermotolerance, the activities of APX, DHAR, MDHAR, GR, CAT, and SOD, as well as corresponding gene expression, were detected. The results indicate that SNP alone significantly increased APX and DHAR activities, and markedly decreased SOD, but had no significant effect on MDHAR, GR, and CAT in maize seedlings under non-HS conditions compared with the CK (**Figures 4**, **5**). In addition, under non-HS conditions, the SNP-induced APX and DHAR activities were reduced by PAG, HA (except for APX), SP, and HT (except for APX) (**Figure 4A, C**). PAG, HA, SP, and HT observably enhanced MDHAR (**Figure 4E**), and markedly lowered CAT and SOD (except for PAG, SP on CAT, and SP on SOD) (**Figures 5A, C**), but there was no significant difference in GR compared with SNP alone (**Figure 4G**). Similarly, under non-HS conditions, SNP alone significantly up-regulated *GR1* expression (**Figure 4H**), but had no significant effect on *APX1*, *DHAR*, *MDHAR1*, *CAT1*, and *SOD4*, compared with the CK (**Figures 4B, D, F**, **5B, D**). In comparison with SNP alone, PAG, HA, SP, and HT significantly down-regulated *APX1* (except for PAG), *CAT1* (except for HT), and *SOD4* (except for PAG and SP) (**Figures 4B**, **5B, D**), but had no significant effect on *DHAR*, *MDHAR1*, and *GR1* (**Figures 4D, F, H**).

Under HS conditions, SNP alone significantly increased the activities of APX, GR, and CAT and decreased DHAR, but had no significant effect on MDHAR and SOD in maize seedlings, compared with CK (**Figures 4**, **5**). Additionally, in comparison to SNP alone, PAG, HA, SP, and HT markedly decreased APX (except for HA), GR, CAT, and SOD activities (**Figures 4A, G**, **5B, D**); observably increased DHAR and MDHAR in maize seedlings (**Figures 4C, E**). For gene expression, SNP alone notably up-regulated the expression of *DHAR* and *GR1* (**Figures 4D, H**), while there was no significant effect on *APX1*, *MDHAR1*, *CAT1*, and *SOD4* in maize seedlings, compared with CK (**Figures 4B, F**, **5B, D**). In comparison to SNP alone, PAG, HA, SP, and HT prominently decreased the expression of *APX1* and *DHAR* (**Figure 4B, D**), whereas a significant effect on *MDHAR1*, *GR1* (except for SP), *CAT1* (except for PAG), and *SOD4* was not observed in maize seedlings (**Figures 4F, H**, **5B, D**). This section’s results indicate that NO and H_2_S crosstalk could activate the enzymatic antioxidant activity in maize seedlings under both non-HS and HS conditions.

### NO and H_2_S crosstalk increases non-enzymatic antioxidants

**Figure 6:**
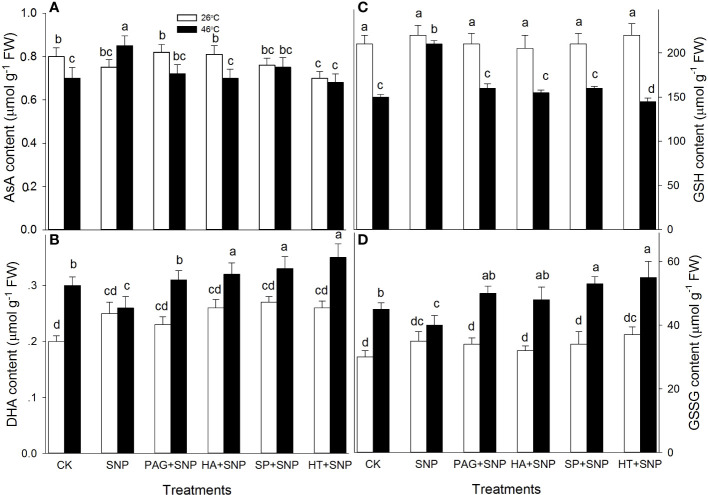
Effect of SNP alone or in combination with hydrogen sulfide (H_2_S) inhibitors and scavengers on ascorbic acid (AsA) **(A)**, dehydroascorbate (DHA) **(B)**, reduced glutathione (GSH) **(C)**, and oxidized GSH (GSSH) **(D)** contents in maize seedlings under non-HS and HS conditions. The data had at least three biological replicates and were tested using Duncan's multiple-range test at 0.05 significant level, which denote means ± standard error (SE). The bars with the different letters represent significant differences, while the same letters represent no significant difference.

**Figure 7:**
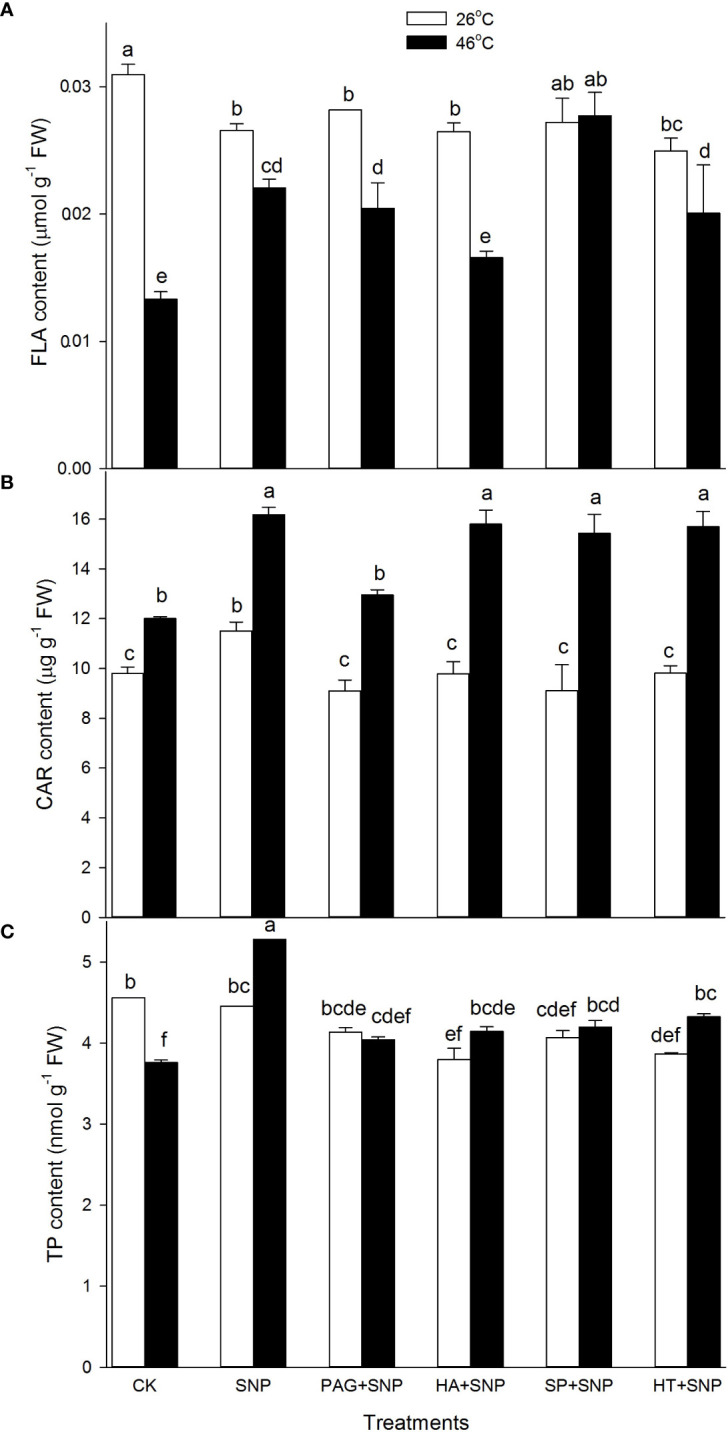
Effect of SNP alone or in combination with hydrogen sulfide (H_2_S) inhibitors and scavengers on flavonoids (FLA) **(A)**, carotenoids (CAR) **(B)**, and total phenols (TP) **(C)** contents in maize seedlings under non-HS and HS conditions. The data had at least three biological replicates and were tested using Duncan's multiple-range test at 0.05 significant level, which denote means ± standard error (SE). The bars with the different letters represent significant differences, while the same letters represent no significant difference.

Besides enzymatic antioxidants, the non-enzymatic antioxidants (AsA, DHA, GSH, GSSG, FLA, CAR, and TP) contents were determined in maize seedlings irrigated with SNP alone or in combination with PAG, HA, SP, or HT. Under non-HS conditions, the results exhibit that SNP alone significantly increased CAR content, and decreased FLA, but had no significant effect on AsA, DHA, GSH, GSSG, and TP in maize seedlings, compared with the CK (**Figures 6**, **7**). In addition, in comparison with SNP alone, PAG, HA, SP, or HT prominently decreased CAR and TP (except for PAG and SP) (**Figures 7B, C**), while the significant effect on AsA, DHA, GSH, GSSG, and FLA was not recorded in maize seedlings (**Figures 6A–D**, **7A**).

Under HS conditions, in comparison with the CK, SNP alone significantly improved AsA, GSH, FLA, CAR, and TP contents, and markedly declined DHA and GSSG in maize seedlings (**Figures 6**, **7**). Compared to SNP alone, PAG, HA, SP, or HT weakened AsA, GSH, and TP contents (**Figures 6A, C**, **7C**), and prominently elevated DHA and GSSG (**Figures 6B, D**), but had no significant effect on FLA (except for a decrease in HA) and CAR (except for a decrease in PAG) in maize seedlings (**Figures 7A, B**). This section’s results indicate that NO and H_2_S crosstalk could increase the non-enzymatic antioxidant level in maize seedlings under both non-HS and HS conditions.

### NO and H_2_S crosstalk weakens H_2_O_2_ and O_2_^.-^

**Figure 8:**
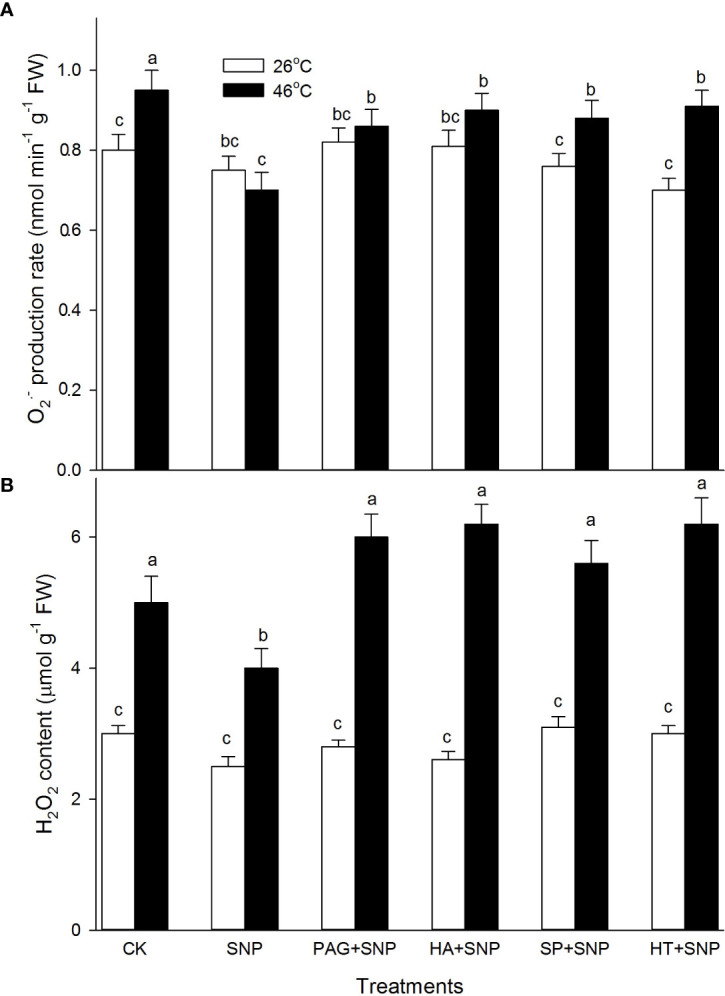
Effect of SNP alone or in combination with hydrogen sulfide (H_2_S) inhibitors and scavengers on superoxide radical (O_2_^.-^) production rate **(A)** and hydrogen peroxide (H_2_O_2_) **(B)** contents in maize seedlings under non-HS and HS conditions. The data had at least three biological replicates and were tested using Duncan's multiple-range test at 0.05 significant level, which denote means ± standard error (SE). The bars with the different letters represent significant differences, while the same letters represent no significant difference.

As described above, the NO and H_2_S crosstalk could enhance the ROS-scavenging system in maize seedlings under both non-HS and HS conditions (**Figures 4**–**7**). To further probe the effect of NO and H_2_S crosstalk on O_2_^.-^ and H_2_O_2_, the levels of ROS in maize seedlings irrigated with SNP alone or in combination with PAG, HA, SP, or HT were detected. As shown in **Figure 8**, under non-HS conditions, SNP alone had no significant effect on the production rate of O_2_^.-^ and the content of H_2_O_2_ compared with CK. Additionally, in comparison with SNP alone, the significant effect of PAG, HA, SP, and HT on ROS levels was not noted in maize seedlings (**Figure 8**). Under HS conditions, compared to the CK, SNP alone significantly decreased O_2_^.-^ (**Figure 8A**) and H_2_O_2_ (**Figure 8B**), compared with SNP alone, PAG, HA, SP, or HT reversed NaHS-decreased O_2_^.-^ (**Figure 8A**) and H_2_O_2_ (**Figure 8B**). These data suggest that NO and H_2_S crosstalk could weaken O_2_^.-^ production rate and H_2_O_2_ levels in maize seedlings under HS conditions.

## Discussion

HS, as a prime abiotic stress factor, adversely affects the whole life cycle of plants from seed germination to organ senescence (Wahid et al., 2007; Hasanuzzaman et al., 2013). The acquirement of plant thermotolerance is an intricate biological process, involving HS sense, signal transduction, gene expression, and physio-biochemical changes (Saidi et al., 2011; Hasanuzzaman et al., 2013; Asthir, 2015; Wani and Kumar, 2020). Signal transduction commonly forms a sophisticated signal network by the crosstalk among signaling molecules, such as Ca^2+^, ROS, NO, H_2_S, and plant hormones (Parankusam et al., 2017; Haider et al., 2021). In this study, under non-HS conditions, SNP alone up-regulated the expression of *LCD1* (**Figure 1B**) and activities of LCD and DCD (**Figures 1A, C**), which in turn increased the endogenous level of H_2_S in maize seedlings (**Figure 1F**). These effects were impaired by cPTIO in maize seedlings, but it was not completely abolished (**Figures 1B, F**), suggesting that other signaling pathways might be in existence. NaHS alone had no significant effect on the gene expression and activity of NR (**Figures 2A, B**). These data hint that the NO could trigger the LCD/H_2_S signaling pathway in maize seedlings under non-HS conditions.

**Figure 9:**
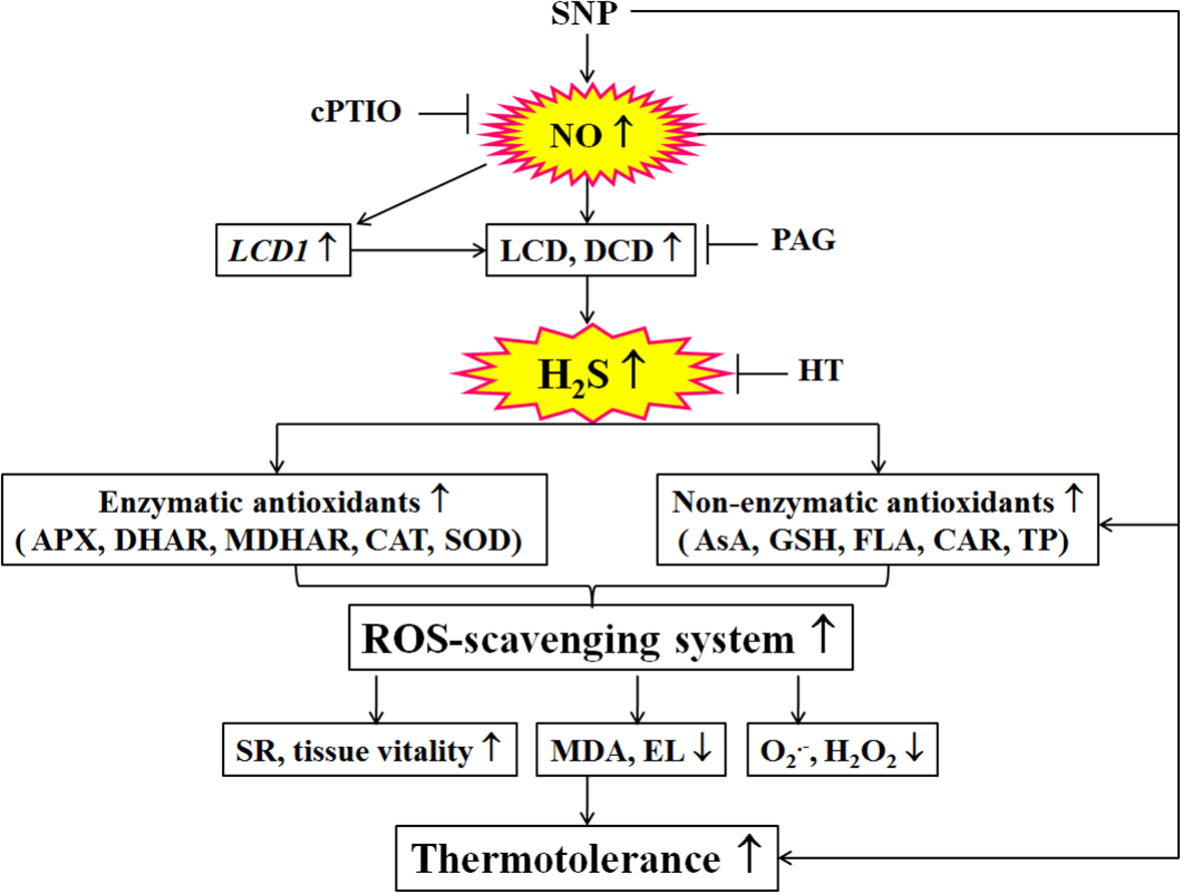
Mechanisms underlying nitric oxide (NO) and hydrogen sulfide (H_2_S) crosstalk evoked thermotolerance in maize seedlings. Nitric oxide (NO) donor sodium nitroprusside (SNP) could trigger hydrogen sulfide (H_2_S) signaling by up-regulated *LCD1* expression and L-cysteine desulfhydrase (LCD) and D-cysteine desulfhydrase (DCD) activities. Then, the activity of the ROS-scavenging system, including enzymatic (ascorbate peroxidase, APX; dehydroascorbate reductase, DHAR; monodehydroascorbate reductase, MDHAR;glutathione reductase, GR; catalase, CAT; and superoxide dismutase, SOD) and non-enzymatic (ascorbic acid, AsA; glutathione, GSH; flavonoids, FLA; carotenoids, CAR; and total phenols, TP) antioxidants, was enhance by SNP, which in turn increased survival rate (SR) and tissue viability, decreased electrolyte leakage (EL), malondialdehyde (MDA), superoxide radical (O_2_^.-^), and hydrogen peroxide (H_2_O_2_) levels, thus evoking thermotolerance of maize seedlings. Also, the SNP-evoked thermotolerance could be weakened by 2-(4-carboxyphenyl)-4,4,5,5- tetrameth- ylimidazoline-1-oxyl-3-oxide (cPTIO), DL-propargylglycine (PGA), and hypotaurine (HT) alone, indicating that ROS-scavenging system plays a key role in the SNP-evoked thermotolerance in maize seedlings. The arrows (↑), (↓), and (⊥) denote increase, decrease, and inhibition, respectively.

Similarly, SNP increased the SR and tissue viability, and reduced MDA and EL in maize seedlings under HS conditions (**Figure 3**), indicating that SNP could evoke the thermotolerance of maize seedlings. SNP-evoked thermotolerance was weakened by PAG and HT alone but was not eliminated (**Figure 3**), similar to the changes in H_2_S triggered by NO (**Figure 1**), suggesting that SNP could evoke thermotolerance in maize seedlings by triggering, at least part of the LCD/H_2_S signaling pathway (**Figure 9**), further supporting the fact that NO cross-talks with H_2_S in maize seedlings under non-HS conditions. In addition, the NO and H_2_S crosstalk-evoked thermotolerance in maize seedlings was supported by a previous study (Li et al., 2013). In maize seedlings, the endogenous level of NO was increased by H_2_O_2_ but abolished by cPTIO (Li et al., 2015). Similarly, H_2_O_2_ alone activated LCD activity, which in turn accumulated endogenous H_2_S, which was diminished by cPTIO (Li et al., 2015). Meanwhile, H_2_O_2_-induced thermotolerance was enhanced by SNP and NaHS alone, but weakened by cPTIO, PAG, and HT, respectively (Li et al., 2015). Analogously, Gautam et al. (2022a) reported that foliar spraying with ethylene could enhance thermotolerance in rice seedlings by cross-talking with H_2_S and NO. In Chinese cabbage, Xie et al. (2018) found that crosstalk between H_2_S and NO existed in the formation of heat tolerance induced by H_2_S and NO alone or in combination. Similar crosstalk between NO and H_2_S could also be seen in Poplar (*Populus trichocarpa*) plants (Cheng et al., 2018). These studies further support our hypothesis that NO and H_2_S crosstalk evoked thermotolerance in plants.

HS usually triggers oxidative stress, biomembrane damage, protein denaturation, osmotic stress, methylglyoxal stress, and so forth (Wahid et al., 2007; Li, 2022). As well as causing damage, oxidative stress is the main cause of HS injury (Asthir, 2015). Therefore, the alleviation of oxidative stress is bound up with the acquisition of plant thermotolerance by enhancing the activity of the ROS-scavenging system. The ROS-scavenging system mainly incorporates enzymatic (e.g. APX, DHAR, MDHAR, GR, CAT, and SOD) and non-enzymatic (e.g. AsA, GSH, FLA, CAR, and TP) antioxidants. APX, DHAR, MDHAR, and GR principally drive the AsA-GSH cycle, which in turn modulates the redox balance in plant cells; while SOD and CAT directly scavenge O_2_^.-^and H_2_O_2_, respectively (Gupta et al., 2018; Hasanuzzaman et al., 2020). Likewise, lipid-soluble non-enzymatic antioxidants (e.g. CAR) quench ROS produced by biomembrane *via* single electron transfer in thylakoids, whereas water-soluble enzymatic antioxidants (e.g. AsA, GSH, FLA, and TP) can eliminate ROS in the cytosol (Bobrovskikh et al., 2020; Sabagh et al., 2021), thus maintaining redox balance in both biomembrane and cytosol to avoid the oxidative damage of plant cells under HS conditions. In this study, under non-HS conditions, SNP alone up-regulated the expression of *GR1* (**Figure 4H**), activities of APX and DHAR (**Figures 4A, C**), and content of CAR (**Figure 7B**), indicating that the SNP-irrigated maize seedlings had a higher activity of ROS-scavenging system. In addition, the expression of genes (*APX1*, *CAT1*, and *SOD4*) (**Figures 4B**, **5B, D**), activities of enzymes (APX, DHAR, CAT, and SOD) (**Figures 4A, C**, **5A, C**), and content of antioxidants (CAR and TP) (**Figures 7B, C**) were down-regulated by PAG, HA, SP, or HT, further supporting the hypothesis that NO and H_2_S crosstalk maintained a higher activity of ROS-scavenging system in maize seedlings under non-HS conditions. Similarly, NO and H_2_S cooperate to enhance thermotolerance in wheat seedlings by reducing glucose sensitivity and oxidative stress *via* the AsA-GSH cycle (Iqbal et al., 2021). Therefore, the enhanced activity of the ROS-scavenging system by NO and H_2_S crosstalk laid the foundation for the acquisition of subsequent thermotolerance in maize seedlings.

In the same way, under HS conditions, the SNP-irrigated maize seedlings maintained higher gene expression of the *DHAR* and *GR1* (**Figure 4D, H**) activities of enzymes (APX, GR, and CAT) (**Figures 4A, G**, **5A**), contents of non-enzymatic antioxidants (AsA, GSH, FLA, CAR, and TP) (**Figures 6**, **7**), and a lower level of O_2_^.-^ and H_2_O_2_ (**Figures 8A, B**), thus improving the thermotolerance in maize seedlings (**Figures 3**, **4**). Adversely, *APX1* and *DHAR* expression (**Figures 4B, D**), enzyme (APX, GR, CAT, and SOD) activities (**Figures 4A, G**, **5B, D**), and non-enzymatic antioxidants (AsA, GSH, and TP) contents (**Figures 6A, C**, **7C**) were weakened by PAG, HA, SP, and HT, followed by maintaining a lower activity of ROS-scavenging system and a higher level of O_2_^.-^ and H_2_O_2_ (**Figures 8A, B**), thus reducing the thermotolerance in maize seedlings (**Figures 3**, **4**). Similarly, in wheat (*Triticum aestivum* L. cv. Irena) seedlings, NO stimulated LCD and DCD activities, which in turn increased the endogenous level of H_2_S, followed by enhancing the activities of GR, POD, SOD, and CAT, thus reducing H_2_O_2_ and O_2_^.-^ levels under osmotic stress (Khan et al., 2017). NO-treated bermudagrass (*Cynodon dactylon* (L). Pers.) had higher H_2_S accumulation, enzymes (SOD, CAT, POD, and GR) activities, and GSH level, compared with the control, therefore maintaining a lower level of ROS (i.e. H_2_O_2_ and O_2_^.-^) (Shi et al., 2014). In addition, the foliar spraying of ethylene enhanced the thermotolerance in rice seedlings by regulating antioxidant enzyme activity, osmolytes, and photosynthetic metabolism *via* crosstalk with NO and H_2_S (Gautam et al., 2022a). These investigations further support the fact that NO and H_2_S crosstalk enhanced the activity of the ROS-scavenging system in plants, suggesting that the ROS-scavenging system played a key role in the NO and H_2_S crosstalk-evoked thermotolerance in maize seedlings. The mechanism underlying NO and H_2_S crosstalk-evoked thermotolerance in maize seedlings is presented in **Figure 9**.

## Conclusions

In summary, NO increased H_2_S level by activating the gene expression of *LCD1* and activities of LCD and DCD, while H_2_S had no significant effect on the gene expression and activity of NR, indicating NO could trigger the LCD/H_2_S signaling pathway in maize seedlings under non-HS conditions. NO evoked thermotolerance in maize seedlings and this evocation was weakened by H_2_S inhibitors and scavengers, further supporting the NO and H_2_S crosstalk in the acquirement of thermotolerance in maize seedlings. Moreover, the vitality of the ROS-scavenging system was enhanced by NO under both non-HS and HS conditions, but it was reduced by H_2_S inhibitors and scavengers, implying the key role of the ROS-scavenging system in the NO and H_2_S crosstalk-evoked thermotolerance in maize seedlings.

## Data availability statement

The original contributions presented in the study are included in the article/supplementary files, further inquiries can be directed to the corresponding author/s.

## Author contributions

Z-GL conceived and designed the experiments and wrote the manuscript. Y-YS performed the experiments. J-QW analyzed the data. R-HX drew the figures. All authors contributed to the article and approved the submitted version.

## Funding

This work was funded by a grant from the National Natural Science Foundation of China (No. 32160065).

## Conflict of interest

The authors declare that the research was conducted in the absence of any commercial or financial relationships that could be construed as a potential conflict of interest.

## Publisher’s note

All claims expressed in this article are solely those of the authors and do not necessarily represent those of their affiliated organizations, or those of the publisher, the editors and the reviewers. Any product that may be evaluated in this article, or claim that may be made by its manufacturer, is not guaranteed or endorsed by the publisher.
